# Oral fibroblasts rescue osteogenic differentiation of mesenchymal stem cells after exposure to Zoledronic acid in a paracrine effect

**DOI:** 10.3389/fphar.2023.1172705

**Published:** 2023-08-11

**Authors:** Tal Berg, Ofri Doppelt-Flikshtain, Benjamin R. Coyac, Hadar Zigdon-Giladi

**Affiliations:** ^1^ Laboratory for Bone Repair, Rambam Healthcare Campus, Haifa, Israel; ^2^ The Ruth and Bruce Rappaport Faculty of Medicine, Technion-Israel Institute of Technology, Haifa, Israel; ^3^ Department of Periodontology, School of Graduate Dentistry, Rambam Health Care Campus, Haifa, Israel

**Keywords:** osteonecrosis, Zoledronic acid, fibroblasts, mesenchymal stem cells, bone

## Abstract

**Background:** Medication-related osteonecrosis of the jaw is a serious complication that develops in oncologic patients treated with Zoledronic acid. Although used for over 30 years, the influence of Zoledronic acid on bone has been thoroughly investigated, mainly on osteoclasts. While decreasing osteoclast differentiation and function, for many years it was thought that Zoledronic acid increased osteoblast differentiation, thus increasing bone volume. Moreover, despite the influence of soft tissue on the bone healing process, the impact of zoledronic acid on the interaction between soft tissue and bone was not investigated.

**Aim:** Our goal was to investigate the influence of Zoledronic Acid and soft tissue cells on osteogenic differentiation of mesenchymal stem cells (MSCs).

**Materials and methods:** Osteogenic differentiation of MSCs was examined after exposure to Zoledronic Acid. To determine the influence of soft tissue cells on MSCs’ osteogenic differentiation, conditioned media from keratinocytes and oral fibroblasts were added to osteogenic medium supplemented with Zoledronic Acid. Proteomic composition of keratinocytes’ and fibroblasts’ conditioned media were analyzed.

**Results:** Zoledronic Acid decreased osteogenic differentiation of MSCs by seven-fold. The osteogenic differentiation of MSCs was restored by the supplementation of fibroblasts’ conditioned medium to osteogenic medium, despite Zoledronic acid treatment. Five osteogenic proteins involved in the TGFβ pathway were exclusively identified in fibroblasts’ conditioned medium, suggesting their role in the rescue effect.

**Conclusion:** Oral fibroblasts secrete proteins that enable osteogenic differentiation of MSCs in the presence of Zoledronic Acid.

## Introduction

Medication-related osteonecrosis of the jaw (MRONJ) is a severe complication of oral infection or oral surgical procedure which develops in patients treated with Zoledronic Acid (ZA). The clinical manifestation of MRONJ includes the presence of exposed bone in the oral cavity for more than 8 weeks, in patients with previous or current use of bone-modifying agents and no history of radiation therapy to the head and neck region (Ruggiero et al., 2022). ZA is considered the most potent drug that causes MRONJ. The occurrence of MRONJ, reported to be 1%–9% in oncologic patients treated with ZA, is relatively high considering that ZA is commonly used to prevent skeletal complications associated with a variety of malignancies, e.g., lung, renal, breast, and prostatic cancers, and multiple myeloma (Yarom et al., 2019).

Surgical resection of the jaw is the main treatment for stage 3 MRONJ and sometimes is implicated in resistant stage 2 cases (Ruggiero et al., 2022). Therefore, prevention of MRONJ is of paramount importance (Yarom et al., 2019; Ruggiero et al., 2022).

Bone remodeling is regulated by a dialogue between osteocytes that reside within the mineralized matrix of bone and cells situated in the soft tissue external or internal to the mineralized matrix, i.e., mesenchymal stem cells (MSCs), osteoblasts, and osteoclasts (Bonewald, 2011). Since first reported in 2003 (Marx, 2003), studies of MRONJ had focused on the inhibitory effect of ZA on osteoclasts via impairing the mevalonic acid pathway (Russell, 2011; Huang et al., 2019; Wang et al., 2020). Soft tissue toxicity is another adverse effect of ZA that has been shown to interfere with bone repair (Reid et al., 2007; Mozzati et al., 2013). Several studies had shown that ZA has toxic effects on epithelial cells, fibroblasts, and endothelial cells (Saracino et al., 2012; Mozzati et al., 2013), thereby interfering with the normal process of wound healing. Scheper et al. (Scheper et al., 2009; Scheper et al., 2010) were the first to show that low concentrations of ZA released from bone can negatively affect the oral mucosal tissues. The exact ZA concentration in the alveolar bone remains unclear. Early studies used 5 μM ZA as the closest concentration to accumulate in the alveolar bone (Scheper et al., 2009) and recent studies use 5μM and 10 μM as subtoxic concentrations (Zara et al., 2015; di Vito et al., 2020).

Our research hypothesis was that ZA impacts the osteogenic differentiation of MSCs but the detrimental effect of ZA is modulated by soft tissue cells, i.e., fibroblasts and keratinocytes. The aim of the study was to investigate the influence of keratinocytes and fibroblasts, the main cellular components of the soft tissue, on MSCs’ osteogenic differentiation after exposure to ZA.

## Materials and methods

### Soft tissue cells culture

#### Fibroblasts cell culture

Three types of primary human oral fibroblasts were used in this study: 1) primary human gingival fibroblasts (ATCC, PCS201-018, Manassas, VA); 2) primary human lining mucosa fibroblasts, which were obtained from the buccal lining mucosa of the anterior mandible; and 3) primary human masticatory mucosa fibroblasts, which were obtained from the hard palate (Kabakov et al., 2021). Cells from passages 3–5 were cultured in a 10 mm plate at a density of 2 × 10^6^ cells per plate (Corning, Glendale, AZ, United States). Cells were cultured in Dulbecco’s Modified Eagle medium (DMEM, Biological Industries, Beit Haemek, Israel) high glucose (gingival fibroblasts) or low glucose (lining/masticatory fibroblasts), supplemented with 10% FBS, 1% penicillin—Streptomycin - Amphotericin B Solution (PSA), then 1% L-Glutamine. Cells were incubated at 37°C and 5% CO_2_.

#### Keratinocytes cell culture

Human Keratinocytes cells (HaCaTs cell line) were seeded at a density of 1 × 10^6^ cells/10 mm plate and cultured in DMEM high glucose supplemented with 10% FBS, 1% PSA, and 1% L-Glutamine until 80% confluence. Cells were incubated at 37°C and 5% CO_2_. HaCats were cultured, and expanded according to a previously published protocol (Gamady et al., 2003; Tamari et al., 2019).

#### Bone cells culture: mesenchymal stem cells culture

Two types of primary human MSCs were used in this study: 1) Primary human bone marrow-derived mesenchymal stem cells (BM-MSCs) (Srouji et al., 2011) and 2) Primary human periodontal ligament derived stem cells (PDL-MSCs) (Somerman et al., 1988). Cells were used from passages 3 to 5 and were cultured in alpha MEM medium (Biological Industries, Beit Haemek, Israel) supplemented with 10% FBS, 1% PSA, and 1% L-Glutamine. Cells were incubated at 37°C and 5% CO_2_.

All growth media types (DMEM High, DMEM Low and alpha MEMα) supplemented with 10% FBS, 1% PSA, and 1% L-Glutamine, are referred as “growth medium” in this study.

### Conditioned medium preparation

1.5 × 10^6^ cells were seeded in a 10 mm culture plate and cultured with 10 mL medium until reaching 80% confluence. Medium was changed to fresh medium and following 24 h incubation 10 mL was collected and stored at −20°C.

### 5 μM ZA solution preparation

190 μL from 4 mg/5 mL ZA (Actavis Italy SpA, Milan, Italy) was diluted in 60 μL growth medium to create stock solution no. 1. 200 μL from stock solution 1 were further diluted in 1,800 mL of growth medium to create stock solution 2. 500μL from stock solution 2 were diluted in 9.5 mL growth medium to create 10 mL of 10 μM ZA solution, and 250 μL from stock solution 2 diluted in 9.5 mL of growth medium created 9.75 mL of 5 μM ZA solution.

### Osteogenic differentiation of MSCs

BM and PDL MSCs were cultured in osteogenic medium composed of alpha MEM medium, supplemented with 10^–7^ M dexamethasone, 5 × 10^−5^ M Ascorbic Acid (Sigma-Aldrich, St. Louis, MO), and 10^−2^mM β Glycerol phosphate disodium salt.

### Analysis of osteogenic differentiation: alkaline phosphatase staining

Following 6 days of culture in osteogenic medium, solutions were replaced by BCIP (5-bromo-4-chloro-3-indolyl phosphate; #11383221001) and NBT (nitro blue tetrazolium chloride; #11383213001; Sigma-Aldrich, St. Louis, MO) which were used as an insoluble substrate for the detection of alkaline phosphatase. Substrate solution was removed, and then the cells were washed with phosphate-buffered saline (PBS) and imaged.

### Analysis of osteogenic differentiation: Alizarin red staining

Following 14 days of culture in osteogenic medium, cells were fixed using 4% paraformaldehyde. A solution of 2% alizarin red (Sigma-Aldrich, St. Louis, MO) was added to each well, and the cells were incubated at 37°C for 30 min in the dark. The alizarin red solution was removed, after which cells were washed four times with dH_2_O and imaged.

### Analysis of osteogenic differentiation: von Kossa reaction

Following 14 days of culture in osteogenic medium, cells in each group were fixed with 4% paraformaldehyde for 15 min at room temperature and washed three times with distilled water. Next, the cells were exposed to 5% silver nitride solution under UV radiation for 30 min at room temperature. The wells were then washed two times with distilled water and observed in a light microscope.

### Preparation of conditioned media mixed with osteogenic media solution supplemented with 5 μM ZA

PDL and BM MSCs were cultured in osteogenic medium for 6 and 14 days. 10 μM ZA solution was prepared as previously described (instead of diluting ZA in growth medium, it was diluted in osteogenic medium) and mixed with soft tissue cells conditioned media in 1:1 volume ratio to achieve a final concentration of 5 μM ZA.

### Quantification of ALP staining, Alizarin red, and von Kossa staining

Each experiment was repeated three times (three different biological repetitions). In each experiment 10 wells were included for each group (technical repeats). Samples were visually inspected using a light microscope (Nikon, Tokyo 108-6290, Japan) using ×4, ×10, ×20 and ×40 objectives. The same setting of color balance, brightness and contrast were used for each image. 15 photographs were taken from each well in ×10 magnification. Quantification made using ImageJ software (National Institute of Health (NIH)) according to their instructions (O’brien et al., 2016).

### Proteomics

Keratinocytes and gingival fibroblasts were cultured in growth medium until 80% confluence. Medium was replaced with starvation medium (0% FBS). After 24 h, 10 mL of the conditioned medium was collected from each type of cells in aliquots of 1 mL and stored at −20°C for proteomics.

### Proteolysis

Medium samples were supplemented with: 8M Urea, 400 mM Ammonium bicarbonate and 10 mM DTT. Protein amount was estimated using Bradford readings. 10ug protein from the sample were reduced with DTT (60°C for 30 min), modified with 8.8 mM iodoacetamide in 400 mM ammonium bicarbonate (in the dark, room temperature for 30 min) and digested in 2M Urea, 25 mM ammonium bicarbonate with modified trypsin (Promega) at a 1:50 enzyme-to-substrate ratio, overnight at 37°C. An additional second digestion with trypsin was done for 4 h at 37°C.

### Mass spectrometry analysis

The eluted peptides were desalted using C18 tips (Homemade stage tips) dried and re-suspended in 0.1% formic acid.

The peptides were resolved by reverse-phase chromatography on 0.075 × 30-mm fused silica capillaries (J&W) packed with Reprosil reversed phase material (Dr Maisch GmbH, Germany). The peptides were eluted with linear 60 min gradient of 5%–28% 15 min gradient of 28%–95% and 15 min at 95% acetonitrile with 0.1% formic acid in water at flow rates of 0.15 μL/min. Mass spectrometry was performed by Q-Exactive plus mass spectrometer (Thermo) in a positive mode (m/z 300–1800, resolution 70,000 for MS1 and 17,500 for MS2) using repetitively full MS scan followed by high collision dissociation (HCD, at 25 normalized collision energy) of the 10 most dominant ions (>1 charges) selected from the first MS scan. The AGC settings were 3e6 for the full MS and 1e5 for the MS/MS scans. The intensity threshold for triggering MS/MS analysis was 1.7e4. A dynamic exclusion list was enabled with exclusion duration of 20 s.

The mass spectrometry data was analyzed using Proteome Discoverer 2.4 software with Sequest (Thermo) algorithms against human database with 1% FDR.

Minimal peptide length was set to six amino acids and a maximum of two mis-cleavages was allowed. Semi quantitation was done by calculating the peak area of each peptide based its extracted ion currents (XICs), and the area of the protein is the average of the three most intense peptides from each protein.

The additional statistical analysis was done by Perseus 1.6.15.0.

### Statistics

Experiments in this study repeated three times to ensure reproducibility. Data were presented as the means ± SE/SD. The significance of the results obtained from control and treated groups were analyzed by GraphPad Prism 9.0 (GraphPad software Inc.) using parametric and non-parametric statistical tests. Differences were considered significant when *p*-value <0.05.

## Results

### ZA significantly reduced osteogenic differentiation of MSCs

**FIGURE 1:**
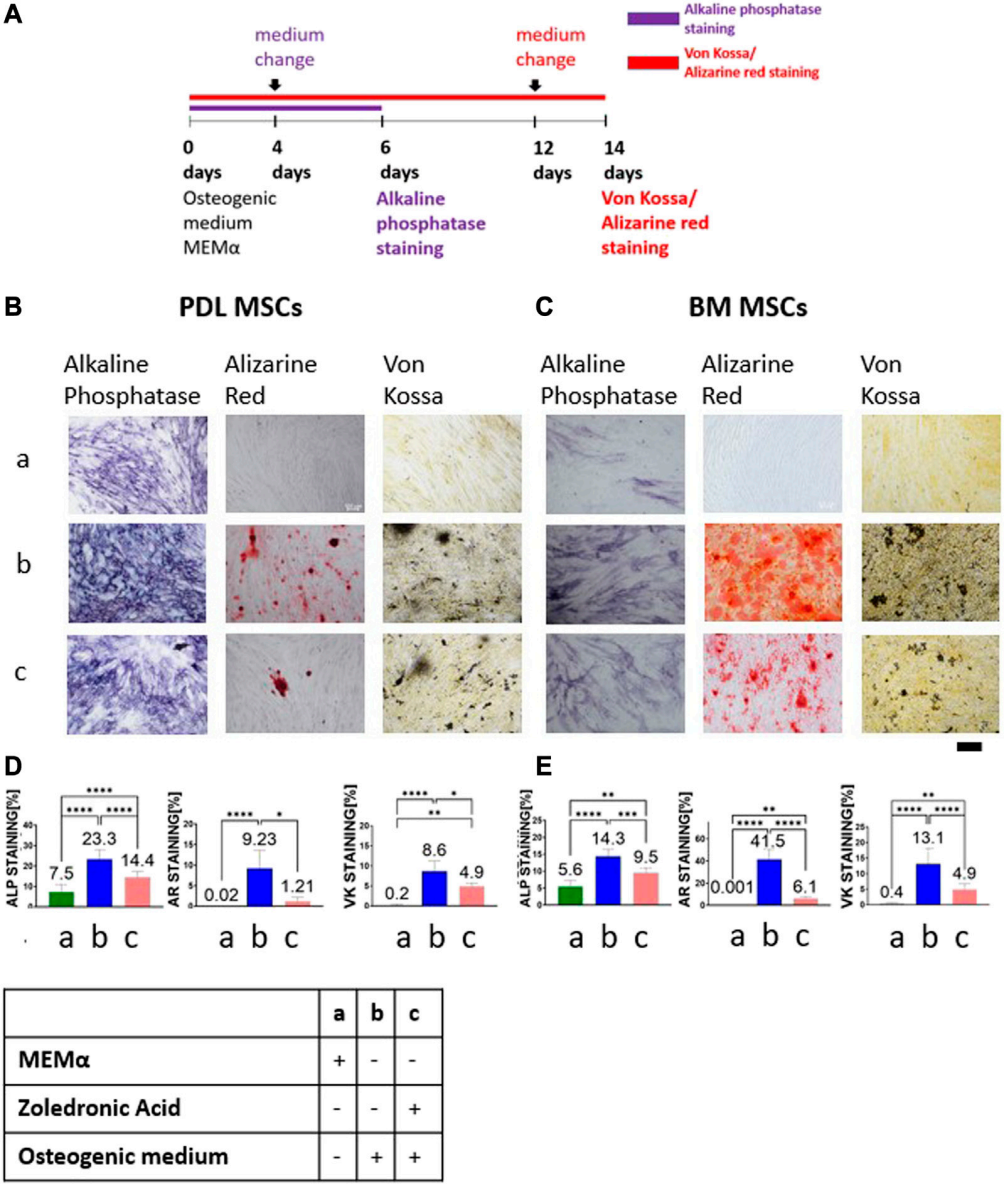
ZA decreased MSCs osteogenic differentiation as detected by alkaline phosphatase staining, Alizarin Red and von Kossa staining. Study timeline is shown in **(A)** Representative light microscope images of PDL and BM MSCs (×10 magnification) with Alkaline phosphatase staining, Alizarin Red and von Kossa staining shown in **(B,C)**. Scale bar represents 400 μm. Statistical analysis of the percentage of the staining for PDL MSCs **(D)** and BM MSCs **(E)**. MEMα showed the weakest staining and osteogenic medium the strongest staining. 5 μM ZA added to osteogenic medium significantly reduced the percentage of the staining as compared to osteogenic medium in both cell types, in all staining methods. Statistical analysis was performed using one way ANOVA and *post hoc* Tukey tests. *p <0.05, **p <0.001, ****p <0.0001.

BM and PDL MSCs were cultured in osteogenic medium and growth medium for 6 days (Figure 1A). On day 4, treatments were changed as follow:1) MEMα as negative control; 2) osteogenic differentiation media as positive control; and 3) 5 μM ZA solution based on osteogenic medium. Figures 1B, C show representative images of the staining from the three groups. The statistical analysis for each of the staining is shown in Figures 1D, E.

ALP staining was quantified on day 6. The weakest ALP staining was demonstrated in the MEMα group (PDL-MSC: 7.5% ± 3.52; BM-MSC:5.6% ± 1.7). The strongest staining was observed in the osteogenic medium group (PDL-MSC: 23.3% ± 4.64; BM-MSC:14.3% ± 2.1). 1:1 5 μM ZA solution based on osteogenic medium group showed a significantly lower percentage staining as compared to the osteogenic medium group (PDL-MSC: 14.4% ± 2.93; BM-MSC:9.5% ± 1.47, *p* <0.0001).

BM and PDL-MSCs were cultured in an osteogenic medium and growth medium for 14 days (Figure 1A). On day 12, medium was changed to: 1) MEMα as negative control; 2) osteogenic differentiation media, positive control; and 3) 1:1 5 μM ZA solution based on osteogenic medium.

The MEMα group showed no Alizarin Red staining (almost 0% in both types of MSCs) as opposed to the osteogenic medium group, which showed the highest percentage staining (PDL-MSC: 9.22% ± 4.35; BM-MSC: 41.5% ± 8.94, *p* <0.0001). The 1:1 5 μM ZA solution based on osteogenic medium group showed significant decrease in staining as compared to osteogenic medium (PDL-MSC: 1.2% ± 0.96; BM-MSC:6.1% ± 1.78, *p* <0.0001). Similarly, no von Kossa staining was detected in the MEMα group for both types of MSCs (PDL-MSC: 0.2% ± 0.22; BM-MSC: 0.4% ± 0.32). The osteogenic medium group showed the highest staining (PDL-MSC: 8.6% ± 2.58; BM-MSC: 13.1% ± 5.08). 1:1 5 μM ZA solution based on osteogenic medium significantly decreased the percentage of staining in both types of MSCs (PDL-MSC: 4.9% ± 0.84; BM-MSC: 4.9% ± 1.83, *p* <0.0001).

### Conditioned medium from primary human gingival fibroblasts restored osteogenic differentiation

**FIGURE 2:**
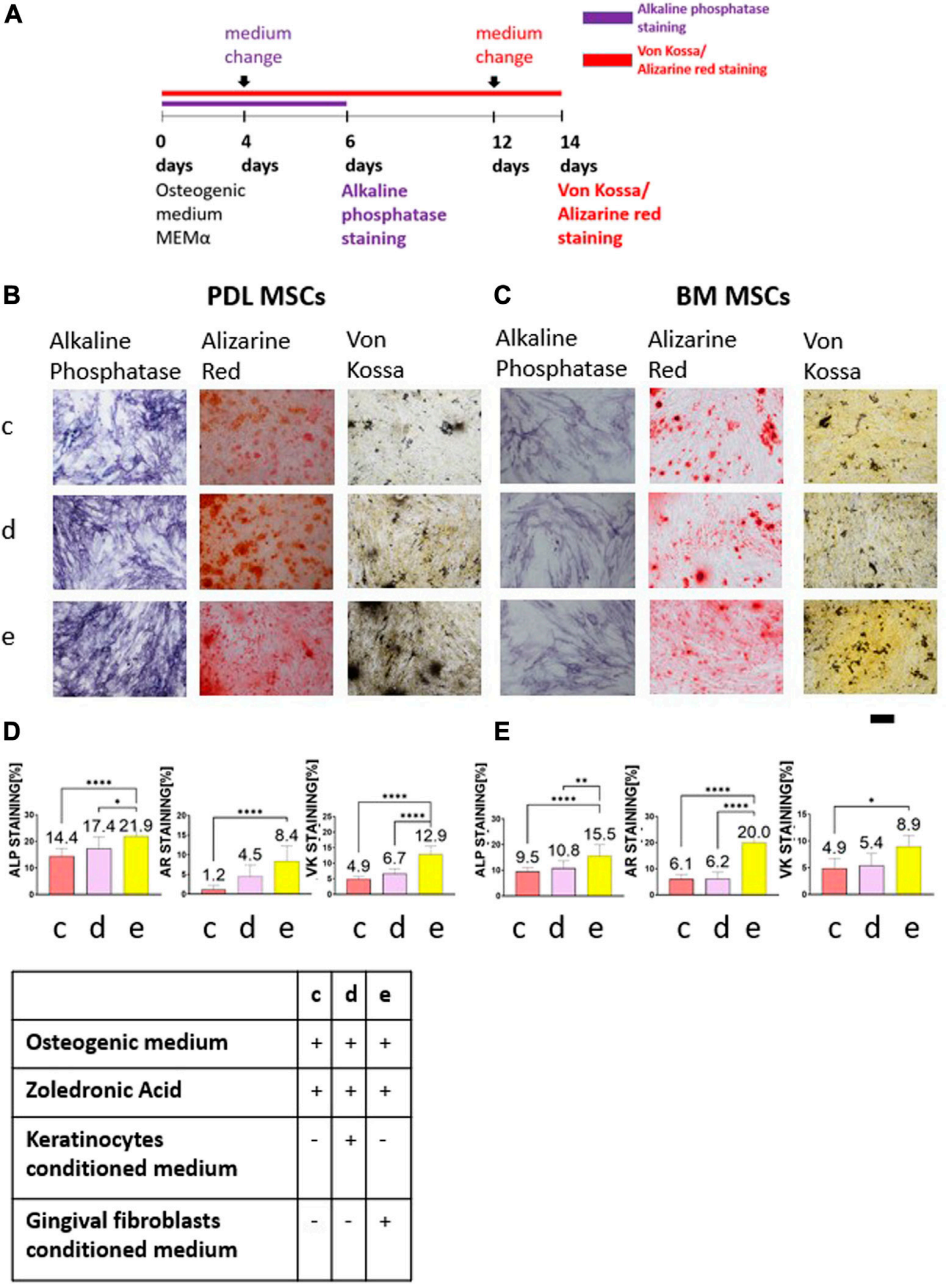
The rescue effect of primary human gingival fibroblasts conditioned medium on osteogenic differentiation after ZA exposure was detected by Alkaline phosphatase staining, Alizarin Red and von Kossa staining. Timeline is shown in **(A)**. Representative light microscope images of PDL **(B)** and BM **(C)** MSCs with Alkaline phosphatase staining, Alizarin Red and Von Kossa staining (×10 magnification). Scale bar represents 400 μm. Statistical analysis of the percentage of the staining for PDL-MSCs **(D)** and BM-MSCs **(E)**. Cells which were exposed to 5 μM ZA solution showed the weakest staining. Keratinocytes conditioned medium did not influence the staining for both MSCs types in all staining methods. Overall, fibroblasts conditioned medium significantly increased the staining. Statistical analysis was performed using one way ANOVA and *post hoc* Tukey tests. *p <0.05, **p <0.001, ****p <0.0001.

BM and PDL MSCs were cultured in osteogenic medium for 6 or 14 days (Figure 2A). Two days before the end of the experiments, medium was changed to: 1) keratinocytes conditioned medium with osteogenic medium and 5 μM ZA solution; 2) gingival fibroblasts conditioned medium with osteogenic medium and 5 μM ZA solution; and 3) osteogenic medium with 5 μM ZA solution. All solutions were prepared in a 1:1 ratio. Figures 2B, C show representative images of the staining from the three groups and the statistical analysis is presented in Figures 2D, E.

The cells cultured in osteogenic medium supplemented with 5 μM ZA solution showed the weakest alkaline phosphatase staining (PDL-MSCs: 14.4% ± 2.93; BM-MSCs:9.5% ± 1.47). The addition of keratinocytes conditioned medium to osteogenic medium supplemented with 5 μM ZA solution did not change the percentage of the staining (PDL-MSC: 17.4% ± 4.32; BM-MSC: 10.8% ± 2.83). The addition of fibroblasts conditioned medium to osteogenic medium supplemented with 5 μM ZA solution significantly increased the percentage of the staining for both cell types (PDL-MSC: 21.9% ± 3.81; BM-MSC:15.5% ± 4.46; *p* <0.0001).

Analysis of Alizarin Red staining after 14 days revealed the lowest percentage of staining in the osteogenic medium supplemented with 5 μM ZA solution group (PDL-MSC: 1.2% ± 0.96; BM-MSC:6.1% ± 1.78). The addition of keratinocytes conditioned medium to the osteogenic medium supplemented with 5 μM ZA solution did not influence the percentage of the staining (PDL-MSC: 4.5% ± 2.86; BM-MSC: 6.2% ± 2.46). However, addition of fibroblasts conditioned medium to the osteogenic medium supplemented with 5 μM ZA solution significantly increased the staining in both types of MSCs (PDL-MSC: 8.4% ± 3.83; BM-MSC: 20% ± 6.22, *p* <0.0001).

The same results were obtained using von Kossa staining. The lowest percentage staining was demonstrated in the osteogenic medium supplemented with 5 μM ZA solution group (PDL-MSC: 4.9% ± 0.84; BM-MSC: 4.9% ± 1.83). The addition of keratinocytes conditioned medium to the osteogenic medium supplemented with 5 μM ZA solution did not influence the staining (PDL-MSC: 6.7% ± 1.57; BM-MSCs: 5.4% ± 2.22). Increase of von Kossa staining was observed in both MSCs types after addition of fibroblasts conditioned medium to the osteogenic medium supplemented with 5 μM ZA solution (PDL-MSC: 12.9% ± 2.52, *p* <0.0001; BM-MSC: 8.9% ± 2.11, *p* <0.05).

### Conditioned medium from primary human lining mucosa and primary human masticatory mucosal fibroblasts restored MSCs osteogenic differentiation

**FIGURE 3:**
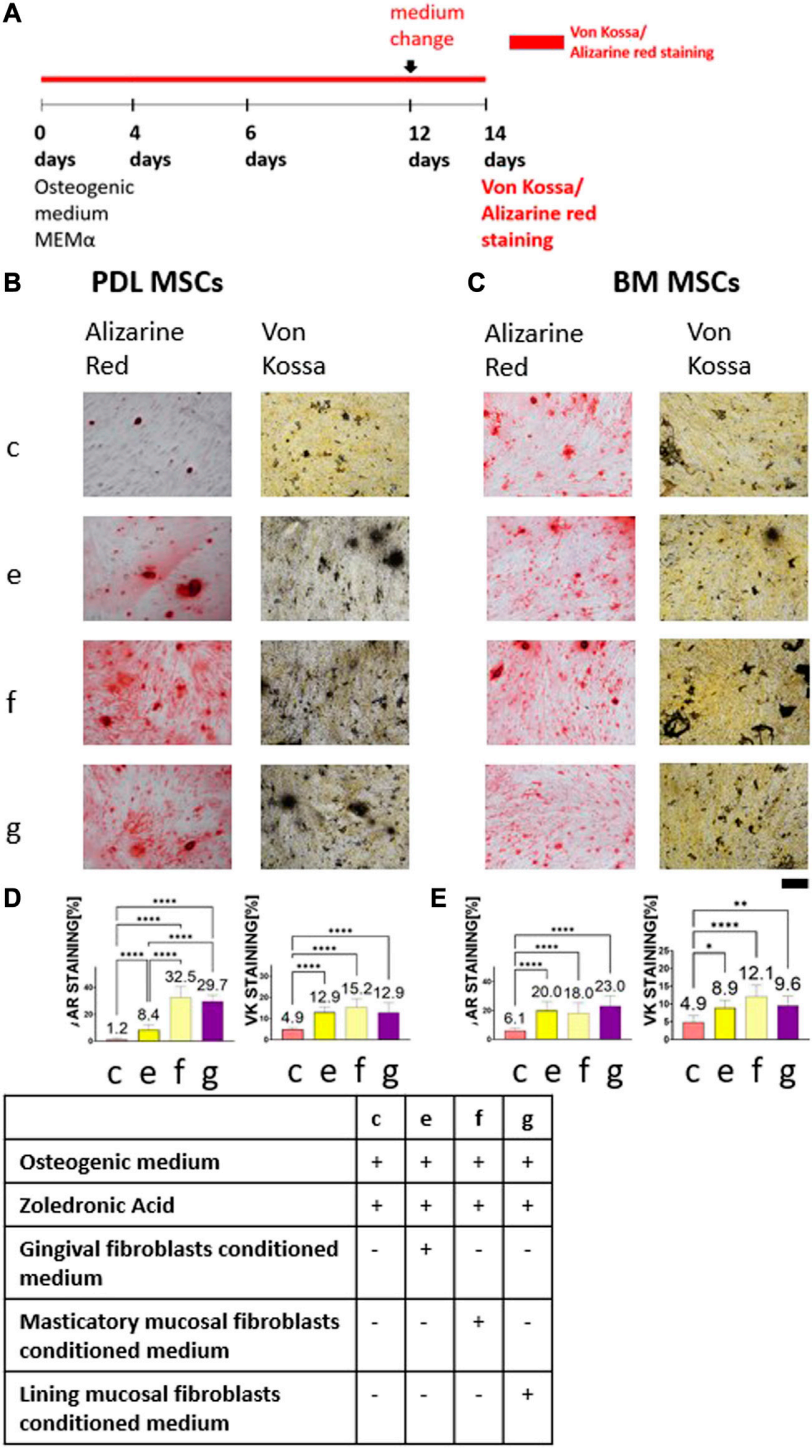
The rescue effect of human gingival fibroblasts conditioned medium on MSCs osteogenic differentiation after ZA exposure was achieved by masticatory mucosal fibroblasts and lining mucosal fibroblasts as detected by Alizarin Red and von Kossa staining. Experiment timeline is shown in **(A)**. Representative light microscope images of PDL **(B)** and BM **(C)** MSCs with Alizarin Red and von Kossa staining (×10 magnification). Scale bar represents 400 μm. Statistical analysis of the percentage of the staining for PDL MSCs **(D)** and BM MSCs **(E)**. Cells which were exposed to 5 μM ZA solution showed the weakest staining in both MSCs types and in all staining methods. Conditioned media obtained from three types of fibroblasts significantly increased the staining. Statistical analysis was performed using one way ANOVA and *post hoc* Tukey tests. *p <0.05, **p <0.001, ****p <0.0001.

Conditioned medium was collected from primary human gingival fibroblasts, primary human masticatory mucosal fibroblasts, and primary human lining mucosal fibroblasts. Conditioned medium from each cell type was mixed with ZA and osteogenic medium to create 5 μM ZA solution. Control group contained cells cultured in 5 μM ZA solution based on osteogenic medium. Figure 3A describes alkaline phosphatase, Alizarin Red and von Kossa staining timeline. Figures 3B, C show representative images of the staining from the four groups: osteogenic medium supplemented with 5 μM ZA solution; human gingival fibroblasts conditioned medium diluted 1:1 with osteogenic medium supplemented with 5 μM ZA solution, masticatory mucosal fibroblasts conditioned medium diluted 1:1 in osteogenic medium supplemented with 5 μM ZA solution and lining mucosal fibroblasts conditioned medium diluted 1:1 with osteogenic medium supplemented with 5 μM ZA solution. The statistical analysis is presented in Figures 3D, E.

The weakest Alizarin Red staining of PDL-MSCs was achieved in the osteogenic medium supplemented with 5 μM ZA solution group (1.2% ± 0.96). Addition of conditioned media from three types of fibroblasts increased Alizarin Red staining: human gingival fibroblasts was 8.4% ± 3.8, *p* <0.0001; masticatory mucosal fibroblasts was 32.5% ± 8.06, *p* <0.0001; and lining mucosal fibroblasts was 29.7% ± 4.55, *p* <0.0001. Likewise, in BM-MSCs the weakest Alizarin Red staining was detected in the osteogenic medium supplemented with 5 μM ZA solution group (6.1% ± 1.78) while addition of fibroblasts’ conditioned medium increased Alizarin Red staining (20% ± 6.22, 18% ± 7.26, and 23% ± 6.22 in human gingival fibroblasts, masticatory mucosal fibroblasts and lining mucosal fibroblasts respectively, *p* <0.0001). Using von Kossa staining, similar results were found. The weakest von kossa staining of PDL-MSCs was achieved in the osteogenic medium supplemented with 5 μM ZA solution group (4.9% ± 0.84). Addition of fibroblasts’ conditioned medium increased von Kossa staining 12.9% ± 2.53, <0.0001; 15.2% ± 4.16, *p* <0.0001; and 12.9% ± 4.53, respectively, *p* <0.0001, in human gingival fibroblasts, masticatory mucosal fibroblasts and lining mucosal fibroblasts respectively. The same pattern was observed in BM-MSCs.

### Conditioned medium from gingival fibroblasts contained specific proteins which are known to induce osteoblastic differentiation

**FIGURE 4:**
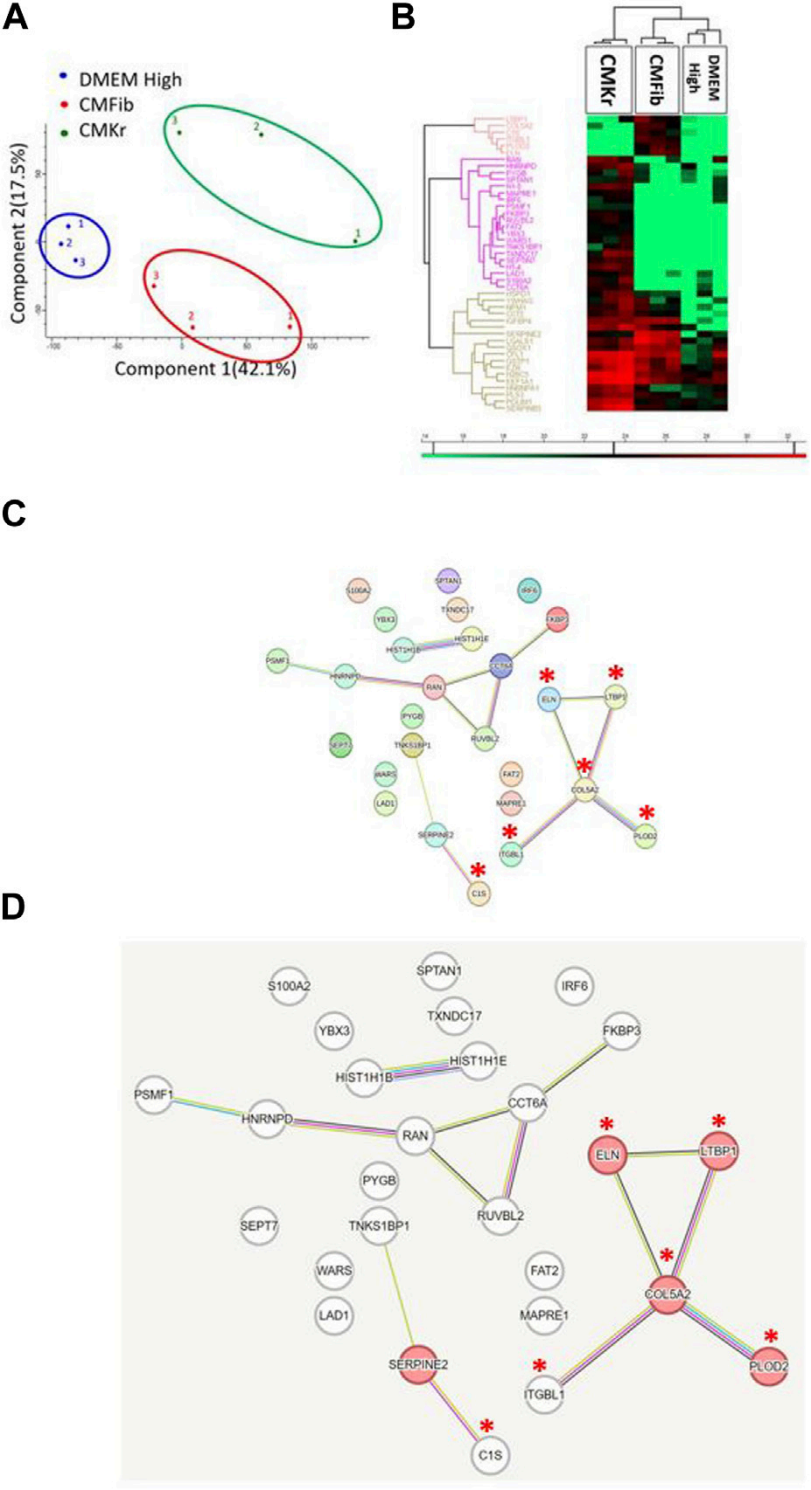
Proteomic analysis: Fibroblasts conditioned medium contains five proteins which are crucial for collagen synthesis and organization. **(A)** Principal Component Analysis (PCA): although CMFib and CMKr present relatively large diversity between the samples, there is still clear separation between the contents of the three groups: DMEM High, CMFib and CMKr. **(B)** Hierarchical clustering of protein abundances resulted in three different clusters containing 44 proteins which were significantly different between CMFib and CMKr. Those proteins are presented in heatmap in which red color presents high abundance and green presents no presence. As expected, most of the proteins were not presented in DMEM High. The first top six proteins were exclusively presented in CMFib. The next 20 proteins were unique to CMKr and the last 18 proteins presented in all solutions. **(C)** STRING functional enrichment analysis was performed to the proteins which showed significant differences in expression between CMFib and CMKr to reveal an interaction network between the proteins. The enlarged figure **(D)** demonstrates the protein network which was composed of the five proteins exclusively presented in CMFib (red asterisks) and are linked to TGFβ signaling (red nodes) as shown by the reference publication analysis performed by the STRING. All five proteins are known to be crucial in collagen synthesis, bone repair, and healing process: Procollagen-lysine,2-oxoglutarate-5-dioxygenase 2 (PLOD2), Integrin β-like 1 (ITGBL1), Collagen 5A (COL5A), latent transforming growth factor β binding protein 1 (LTBP1), and Elastin (ELN) (CMKr, keratinocytes conditioned medium; CMFib, human gingival fibroblasts conditioned medium).

Conditioned media from primary human gingival fibroblasts and keratinocytes were collected after 24 h of culture in starvation. For each type of cells, three samples from different cell passage were used for proteomic analysis (Figure 4). A principal component analysis of the individual samples revealed that DMEM High, fibroblasts conditioned medium, and keratinocytes conditioned medium were separated into three distinct groups (Figure 4A). This was also the case for a hierarchical cluster analysis of protein abundances (Figure 4B), which resulted in three clusters containing 44 proteins in which abundances were distinct between the two conditioned media. The first top six proteins were exclusively presented in the fibroblasts conditioned medium. The next twenty proteins were unique to keratinocytes conditioned medium and the last eighteen proteins were presented in all solutions. A STRING functional enrichment analysis of the proteins which showed significant differences in expression between fibroblasts conditioned medium and keratinocytes conditioned medium (excluding proteins in DMEM) was performed to reveal an interaction network between the proteins. Proteins which were found exclusively in the fibroblasts conditioned medium are marked with red asterisks (Figure 4C). The red nodes represent proteins which participate in the TGFβ signaling according to the reference publication analysis performed by the STRING (Figure 4D) (Szklarczyk et al., 2019). Protein exclusively found in fibroblasts conditioned medium include Procollagen-lysine, 2-oxoglutarate-5-dioxygenase 2 (PLOD2), Integrin β-like 1 (ITGBL1), collagen 5A (COL5A), latent transforming growth factor β binding protein 1 (LTBP1), and Elastin (ELN). All five proteins are known to be related to collagen synthesis and organization during bone remodeling and healing process.

## Discussion

MRONJ is a severe complication of oncologic patients who receive high dosage of ZA. In the last 20 years, the pathobiology of MRONJ has focused on the striking osteoclast inhibition by ZA (Russell, 2011). However, its influence on all actors of bone homeostasis had not been studied until now. For years it was speculated that ZA stimulates osteoblasts’ proliferation and inhibits osteocytes apoptosis or does not influence osteoblasts at all (Rogers et al., 2000; Maruotti et al., 2012; Chen et al., 2018).

Although not the first time that an inhibiting effect of 5 μM ZA on MSCs is demonstrated (di Vito et al., 2020), our study highlights the critical role of the surrounding soft tissue, specifically fibroblasts, in maintaining osteogenic differentiation in the presence of ZA.

As on MSCs and osteoblasts, the effect of ZA on soft tissue cells viability is also dose dependent (Scheper et al., 2009; Scheper et al., 2010). 1 μM ZA is considered a threshold concentration above which osteoblastic proliferation and differentiation is impaired (Zara et al., 2015; di Vito et al., 2020). The impact on soft tissue cells increases as concentrations rise. It significantly impaired soft tissue cells function in a wound scratch assay (Yuan et al., 2019).

BM MSCs are considered the gold standard for *in vitro* studies of bone differentiation. PDL MSCs present similar characteristics and demonstrate an osteogenic differentiation potential as well (Choi et al., 2011; Liu et al., 2014; Abedian et al., 2020). MSCs produce ALP shortly after the beginning of their osteogenic differentiation. Hence, the ALP activity reaction represents the first stages of osteogenic differentiation. Alizarin Red is a low cost and easy staining which demonstrate calcium nodules, which appear at the mineralization stage of the osteogenic differentiation. However, Alizarin Red tend to be washed easily from the well, and Von Kossa reaction which also detects calcium deposits was used to confirm the findings after Alizarin Red staining. Using all methods verified that the osteogenic differentiation could be completed *in vitro* and strengthened our results (Hashemibeni et al., 2013; Lee et al., 2017; Jeon et al., 2018).

Our study showed a smaller osteogenic differentiation potential of PDL MSCs, which exhibited less mineral deposition as demonstrated in weaker Alizarin Red and von Kossa staining. This finding is consistent with another study which found that in addition to weaker Alizarin Red and alkaline phosphatase staining, there was also lower expression of alkaline phosphatase and RunX2 in gene and protein levels (Liu et al., 2014). A 5 μM ZA solution significantly decreased the osteogenic differentiation in the initial and mineralization stages. It is possible that ZA decreased the expression of key markers genes, such as alkaline phosphatase and RunX2 which mark the initial phase and Osteocalcin which marks the mineralization phase (Choi et al., 2011; Basso et al., 2013). Moreover, the most significant finding was that the addition of fibroblasts’ conditioned medium to MSCs with ZA reversed this effect and enabled osteogenic differentiation. This finding demonstrated a paracrine effect of fibroblasts on osteoblasts progenitor cells.

Masticatory mucosal fibroblasts significantly increased PDL MSCs osteogenic differentiation as compared to gingival fibroblasts, as detected by Alizarin Red staining. There was also an increase in differentiation as compared to lining mucosal fibroblasts, although not statistically significant. Masticatory mucosal fibroblasts exhibit a genetic profile which enables a higher degree of plasticity as compared to lining mucosal fibroblasts (Kabakov et al., 2021).

The ability of human gingival fibroblasts secretome to accelerate wound healing through its anti-inflammatory and pro-angiogenic content has already been studied (Ahangar et al., 2020). Whereas keratinocytes express genes, essential for their role as mucosal barrier, fibroblasts express unique genes which are related to the extracellular matrix (Lee et al., 2013). Similarly, our proteomic analysis found five exclusive proteins in fibroblasts conditioned medium which are critical to collagen synthesis. Collagen5A (COL5A) is highly involved in the formation of both endochondral and intramembranous ossification (Wu et al., 2010). Procollagen-lysine,2-oxyglutarate and 5-dioxygenase (PLOD2) were demonstrated to be specifically upregulated during the late stage of osteoblastic differentiation *in vitro* (Uzawa et al., 1999). Integrinβ-like 1 (ITGBL1) is highly expressed in bone remodeling via TGFβ pathway and mediation of RunX2 activation (Li et al., 2015). Latent transforming growth factor β (LTBP1) regulates the bioactivity of TGFβ (Dallas et al., 2000; Dallas et al., 2002). In addition, a recent study showed its crucial role in mandibular growth process (Xiong et al., 2020). Elastin (ELN) was found to have the greatest power to predict *in vivo* bone formation (Twine et al., 2014). All five proteins, exclusively found in fibroblasts’ conditioned medium, are known to be connected to bone repair via the TGFβ signaling pathway as presented by the STRING analysis. TGFβ is an important bone differentiation inducer, and its altered levels were observed in impaired wound healing *in vitro* and *in vivo* (Kim et al., 2017; Berberich et al., 2020).

## Conclusion

Our study confirmed paracrine effect of fibroblasts on osteoblasts progenitors, which rescued osteogenic differentiation after exposure to ZA. More study is needed to explore the influence of direct contact between fibroblasts and osteoblasts progenitors. Moreover, we present an *in vitro* study, which is very limited. Several *in vivo* MRONJ models showed preventive and therapeutic effects of MSCs and endothelial progenitor cells conditioned media on MRONJ (Kikuiri et al., 2010; Matsuura et al., 2016; Doppelt et al., 2020). MSCs, and to much greater extent endothelial progenitor cells, are very difficult to obtain and expand because of their low numbers in blood and bone marrow. Fibroblasts are easily extracted from oral mucosa and the procedure of a connective tissue graft, which can be used to seal a socket after an extraction and is commonly performed in periodontology. Therefore, the findings in this study should be examined in an *in vivo* model to confirm its clinical implications.

## Clinical relevance

### Scientific rationale for study

Medication Related Osteonecrosis of the Jaw (MRONJ) occurs mainly in oncologic patients and can lead to severe mutilation, which dramatically decreases quality of life. As a result, dentists are reluctant to perform necessary procedures in high-risk patients.

### Principal findings

Our study found the ability of fibroblasts’ secretome to rescue osteogenic differentiation of MSCs is probably due to its unique content, which included pro-osteogenic proteins.

### Practical implications

Connective tissue grafts, which contain oral fibroblasts, can be used to seal extraction socket in order to induce bone differentiation, thus promoting healing and preventing MRONJ.

## Data Availability

The raw data supporting the conclusion of this article will be made available by the authors, without undue reservation.

## Abbreviations

ZA, zoledronic acid; MRONJ, medication related osteonecrosis of the jaw; MSCs, mesenchymal stem cells; PDL, periodontal ligament; BM, bone marrow; CMFib, fibroblasts conditioned medium; CMKr, keratinocytes conditioned medium.
