# Supplementary figures and images for: Oral fibroblasts rescue osteogenic differentiation of mesenchymal stem cells after exposure to Zoledronic acid in a paracrine effect
Source: Front Pharmacol. 2023 Aug 11;14:1172705. doi: 10.3389/fphar.2023.1172705 (PMC10450747; doi:10.3389/fphar.2023.1172705)

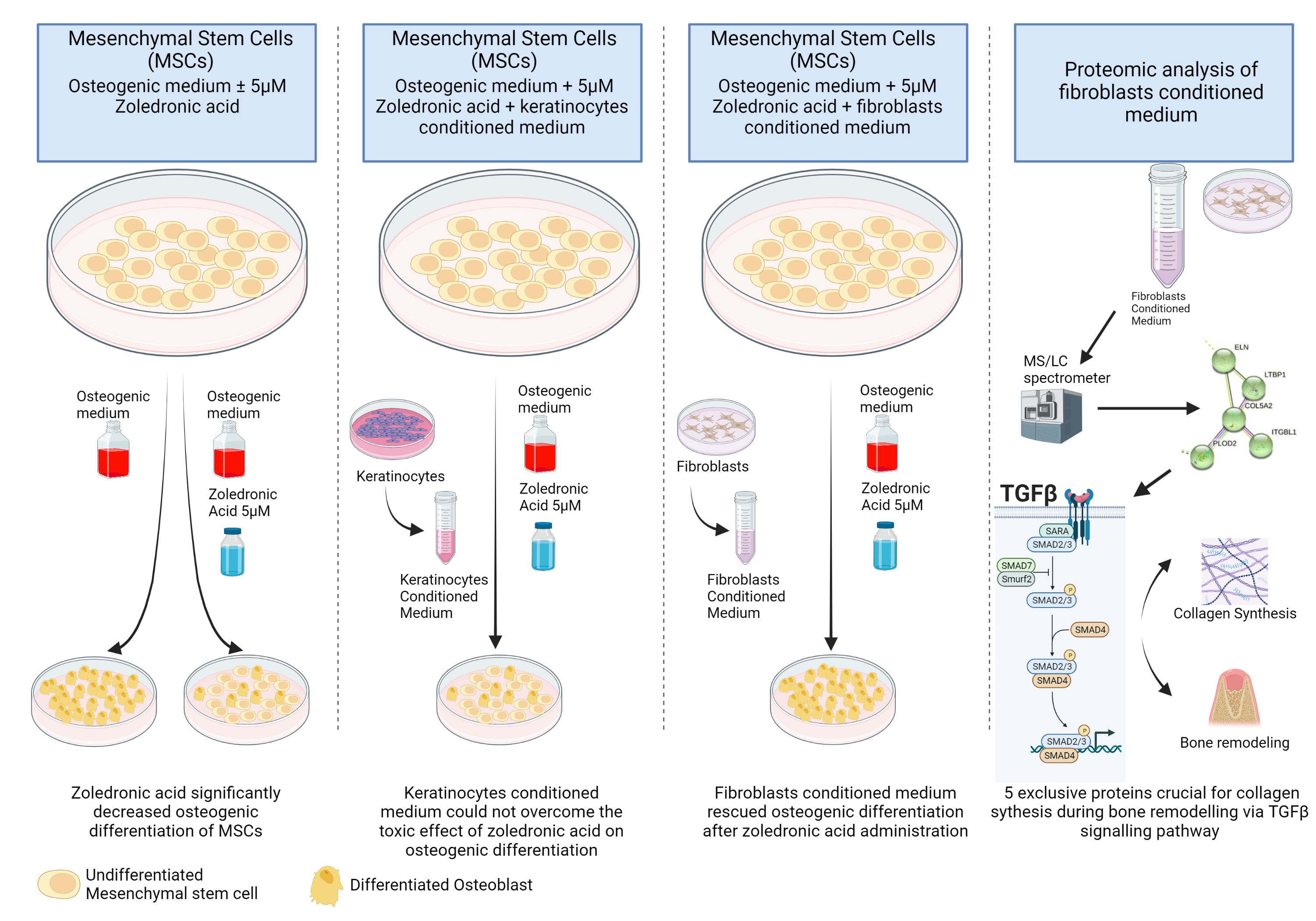

Supplement: Supplementary file 1 [file Image1.PNG]
